# Effects of 5-aminolevulinic acid supplementation on home-based walking training achievement in middle-aged depressive women: randomized, double-blind, crossover pilot study

**DOI:** 10.1038/s41598-018-25452-2

**Published:** 2018-05-08

**Authors:** Hiroshi Suzuki, Shizue Masuki, Akiyo Morikawa, Yu Ogawa, Yoshi-ichiro Kamijo, Kiwamu Takahashi, Motowo Nakajima, Hiroshi Nose

**Affiliations:** 1 Department of Sports Med. Sci., Shinshu University Grad. Sch. of Med., Matsumoto, 390-8621 Japan; 2 Institute for Biomed. Sci., Shinshu University, Matsumoto, 390-8621 Japan; 3 Aoba Kokoro-no Clinic, Tokyo, 170-0002 Japan; 4 Aoba Promotion Co., Ltd., Tokyo, 170-0002 Japan; 5 Department of R&D, SBI Pharmaceuticals Co., Ltd., Tokyo, 106-6020 Japan

## Abstract

Depressive patients often experience difficulty in performing exercise due to physical and psychological barriers. We examined the effects of 5-aminolevulinic acid (ALA) with sodium ferrous citrate (SFC) supplementation during home-based walking training in middle-aged depressive women. Nine outpatients [53 ± 8 (SD) yr] with major depressive disorder participated in the pilot study with randomized, placebo-controlled, double-blind crossover design. They underwent two trials for 7 days, each performing interval walking training (IWT) with ALA + SFC (ALA + SFC) or placebo supplement intake (PLC) intermittently with >a 10-day washout period. For the first 6 days of each trial, exercise intensity for IWT was measured by accelerometry. Before and after each trial, subjects underwent a graded cycling test, and lactate concentration in plasma ([Lac^−^]_p_), oxygen consumption rate (${\dot{{\bf{V}}}{\bf{O}}}_{{\bf{2}}}$), and carbon dioxide production rate (${\dot{{\bf{V}}}{\bf{\text{CO}}}}_{{\bf{2}}}$) were measured with depression severity by the Montgomery–Åsberg Depression Rating Scale (MADRS). We found that the increases in [Lac^−^]_p_, ${\dot{{\bf{V}}}{\bf{O}}}_{{\bf{2}}}$ and ${\dot{{\bf{V}}}{\bf{\text{CO}}}}_{{\bf{2}}}$ during the test were attenuated only in ALA + SFC ([before vs. after] × workload; all, P < 0.01), accompanied by increased training days, impulse, and time at fast walking during IWT (all, P < 0.05) with decreased MADRS-score (P = 0.001). Thus, ALA + SFC supplementation increased IWT achievement to improve depressive symptoms in middle-aged women.

## Introduction

Recently, the number of depressive patients has been increasing in Japan; the life-time prevalence is 6.7%, and women are affected twice as frequently as men^1^. Some depressed people feel unhappy and sad, while others have difficulty doing anything: going out, dressing, face-washing, and taking a bath^2^. One of their primary symptoms is fatigability, resulting in physical inactivity, reducing physical fitness, which would contribute to lifestyle-related diseases, including depression, falling into a viscous cycle^3,4^.

To prevent this, habitual exercise with moderate or higher intensity for ~30 min per day for 3–4 days per week has been recommended^5–10^. However, it may be difficult for depressive patients to perform exercise at the desired intensity, frequency and duration due to physical and psychological barriers^11–14^.

On the other hand, Masuki *et al*.^15^ recently examined the effects of oral supplementation of 5-aminolevulinic acid (ALA) + sodium ferrous citrate (SFC), a precursor of haeme that composes complex IV in the mitochondrial electron transport chain (ETC)^16,17^, on the voluntary achievement of high-intensity interval walking training (IWT) for 6 days in older women without depression and suggested that the achievement of the fast walking training time was enhanced by 69% in the experimental trial compared with the placebo trial. IWT is a home-based walking training programme that repeats more than 5 sets of fast and slow walking for 3 min each per day, which is equivalent to more than 70% and 40% of peak aerobic capacity ($\dot{{\rm{V}}}$O_2 peak_), respectively^18–20^. Regarding the mechanisms, since increases in oxygen consumption rate (${\dot{{\rm{V}}}{\rm{O}}}_{2}$), carbon dioxide production rate (${\dot{{\rm{V}}}\text{CO}}_{2}$) and plasma lactate concentration ([Lac^−^]_p_) were reduced at a given exercise intensity during the graded cycling exercise in the experimental trial but not in the placebo trial after the intervention, these findings suggest that the higher achievement of IWT was attained by a reduced increase in [Lac^−^]_p_ with improved oxygen utilization efficiency to produce adenosine-tri-phosphate (ATP) by activating ETC to improve subjective feeling against exercise stress.

Accordingly, we hypothesized that ALA + SFC supplementation would increase the achievement of IWT by lowering barriers to higher intensity exercise in depressive patients and thereby improving their symptoms.

## Results

### Subject characteristics

**Figure 1:**
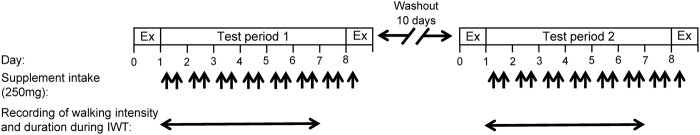
Experimental protocol. The study was conducted using a randomized, placebo-controlled, double-blind crossover design. Ex, graded cycling exercise test and depression severity assessment; IWT, high-intensity interval walking training. In each instance of supplement intake indicated by arrows, subjects ingested 250 mg of either 5-aminolevulinic acid (ALA) + sodium ferrous citrate (SFC) in the ALA + SFC trial or placebo supplement in the PLC trial at breakfast and at dinner (250 mg × 2 = 500 mg/day). See Supplemental Table S1 for details on the supplement compositions.

Subject characteristics, clinical history of depression, and current medications are shown in Table 1. As shown in Table 2, body weight, blood pressure at rest, peak heart rate (HR_peak_)_,_ and ${\dot{{\rm{V}}}{\rm{O}}}_{2}$_peak_ remained unchanged after the supplement intake in both trials (all, *P* > 0.08)(see Fig. 1 for experimental protocol). Resting heart rate (HR_rest_) decreased significantly after the ALA + SFC supplement intake (ALA + SFC trial) (*P* = 0.0014) but not after the placebo supplement intake (PLC trial) (*P* = 0.36). Haemoglobin concentration [Hb] decreased significantly in both trials after the supplement intake period (*P* < 0.05), with no significant interactive effects of [trials x (before vs. after)] (*P* > 0.7).

**Table 1:** Physical characteristics, clinical history, and current psychological drug used for therapy in individual subjects.

Subject No.	Age, yr	Height, cm	BMI, kg/m^2^	Clinical history of depression, mo	Psychotropic drugs
1	58	159.9	16.8	31	Ethyl loflazepate
2	42	151.4	24.0	36	Paroxetine, Ethyl loflazepate, Rilmazafone
3	47	155.1	30.5	55	Paroxetine, Trazodone, Bromazepam, Zopiclone
4	61	159.7	24.0	4	Ethyl loflazepate
5	65	154.1	16.8	24	Ethyl loflazepate
6	52	154.8	29.4	48	Fluvoxamine, Trazodone, Bromazepam, Zolpidem
7	42	166.5	37.2	36	Paroxetine, Mirtazapine, Trazodone, Sulpiride, Ethyl loflazepate, Zolpidem
8	55	158.1	22.9	54	Sertraline, Zolpidem
9	56	154.2	23.3	30	Duloxetine, Ethyl loflazepate, Lorazepam, Clonazepam, Pregabalin, Brotizolam
Mean ± SD	53 ± 8	157.1 ± 4.5	25.0 ± 6.5	35 ± 16	

BMI, body mass index.

**Table 2:** Physical characteristics of subjects.

	PLC		ALA + SFC	
	Before	After	Before	After
Body weight, kg	61.9 ± 6.2	61.7 ± 6.1	62.0 ± 6.3	62.1 ± 6.2
SBP_rest_, mmHg	116 ± 3	123 ± 4	120 ± 2	116 ± 4
DBP_rest_, mmHg	80 ± 5	78 ± 4	74 ± 5	77 ± 4
HR_rest_, beats/min	76 ± 3	78 ± 4	78 ± 3	73 ± 3**
HR_peak_, beats/min	153 ± 2	156 ± 2	157 ± 3	156 ± 2
${\dot{{\rm{V}}}{\rm{O}}}_{2}$_peak_, l/min	1.53 ± 0.09	1.58 ± 0.10	1.54 ± 0.09	1.56 ± 0.09
[Hb], g/dl	12.6 ± 0.2	12.3 ± 0.2*	12.7 ± 0.2	12.4 ± 0.4*

Values are the means ± SE for 9 subjects in each trial. PLC, placebo intake condition; ALA + SFC, 5-aminolevulinic acid + sodium ferrous citrate intake condition; SBP_rest_ and DBP_rest_, resting systolic and diastolic blood pressure, respectively; HR_rest_, resting heart rate; HR_peak_, peak heart rate at $\dot{{\rm{V}}}$O_2 peak_; $\dot{{\rm{V}}}$O_2 peak_, peak oxygen consumption rate during the graded cycling test; [Hb], haemoglobin concentration in blood. *,** Compared with before supplement intake, *P* < 0.05 and *P* < 0.01, respectively.

### ${\dot{{\bf{V}}}{\bf{O}}}_{{\bf{2}}}{\boldsymbol{,}}{\dot{{\bf{V}}}{\bf{\text{CO}}}}_{{\bf{2}}}$, and [Lac^−^]p during the graded cycling test

**Figure 2:**
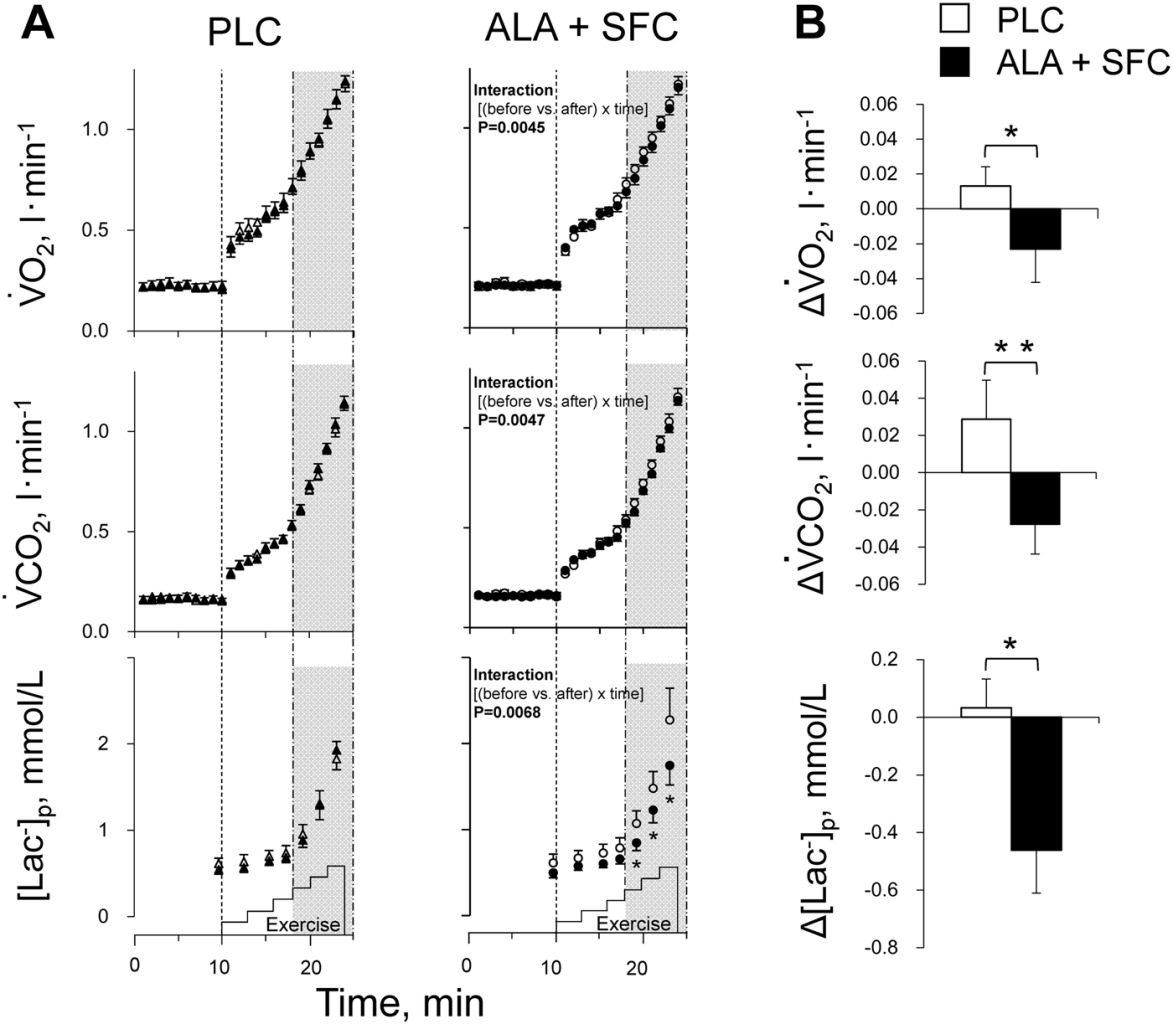
(**A**) Oxygen consumption rate (${\dot{{\rm{V}}}{\rm{O}}}_{2}$), carbon dioxide production rate (${\dot{{\rm{V}}}\text{CO}}_{2}$), and plasma lactate concentration ([Lac^−^]_p_) responses over intensities during the graded cycling exercise test in the PLC trial (*left*) and in the ALA + SFC trial (*right*). The average value per minute in ${\dot{{\rm{V}}}{\rm{O}}}_{2}$ and ${\dot{{\rm{V}}}\text{CO}}_{2}$, and the values at every intensity of [Lac^−^]_p,_ from rest to the highest workload of 75 W, at which all subjects could maintain the rhythm of pedalling at 60 cycles/min. Open symbols, before a supplement intake period; solid symbols, after a supplement intake period. Values are the means ± SE for 9 subjects. *P < 0.05 vs. before the supplement intake period. (**B**) The average changes in ${\dot{{\rm{V}}}{\rm{O}}}_{2}$, ${\dot{{\rm{V}}}\text{CO}}_{2}$, and [Lac^−^]_p_ over intensities ≥45 W during graded cycling exercise after a supplement intake period. Values are the means ± SE for 9 subjects. *P < 0.05 and **P < 0.01 between the PLC and ALA + SFC trials.

As shown in Fig. 2A, although ${\dot{{\rm{V}}}{\rm{O}}}_{2},\,{\dot{{\rm{V}}}\text{CO}}_{2}$, and [Lac^−^]p increased similarly as the intensity increased during the graded cycling test before the supplement intake period in both trials, their increases were significantly attenuated in the ALA + SFC trial after the period with significant interactive effects of [(before vs. after) × time] (*P* = 0.0045, 0.0047, and 0.0068; 1-β = 0.978, 0.978, and 0.906, respectively) but not in the PLC trial (all, *P* > 0.2). Comparing the results before and after the period, [Lac^−^]_p_ decreased significantly at every intensity ≥45 W (*P* = 0.02, 1-β = 0.71) in the ALA + SFC trial but not in the PLC (*P* > 0.7). There was no significant change in ventilation volume ($\dot{{\rm{V}}}$_E_) response before and after the period (*P* > 0.4).

Figure 2B shows the average changes in ${\dot{{\rm{V}}}{\rm{O}}}_{2}$, ${\dot{{\rm{V}}}\text{CO}}_{2}$, and [Lac^−^]_p_ over the intensities ≥45 W during the graded cycling test after the supplement intake period; these values were determined by subtracting the pre-supplementation values from the post-supplementation values at every minute (${\dot{{\rm{V}}}{\rm{O}}}_{2}$, ${\dot{{\rm{V}}}\text{CO}}_{2}$) and every intensity ([Lac^−^]_p_) ≥45 W in each subject, averaging them over the intensities, and presenting the mean and SE values for 9 subjects in each supplement trial. There were significant differences in the changes in ${\dot{{\rm{V}}}{\rm{O}}}_{2}$, ${\dot{{\rm{V}}}\text{CO}}_{2}$, and [Lac^−^]_p_ between trials (all, *P* < 0.03, 1-β > 0.63).

### Training achievement during the supplement intake period

**Figure 3:**
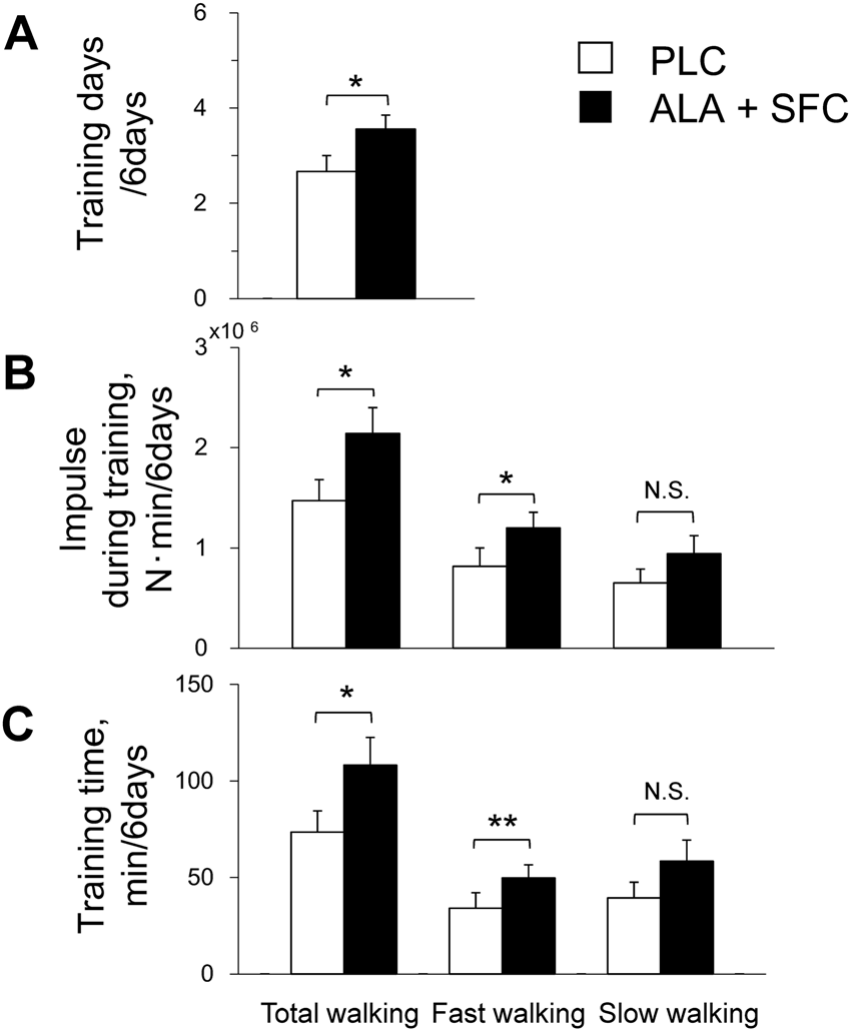
Training days (**A**), training impulse (**B**), and training time (**C**) for total, fast, and slow walking during a supplement intake period. Values are the means ± SE for 9 subjects. *P < 0.05 and **P < 0.01 between the PLC and ALA + SFC trials.

**Figure 4:**
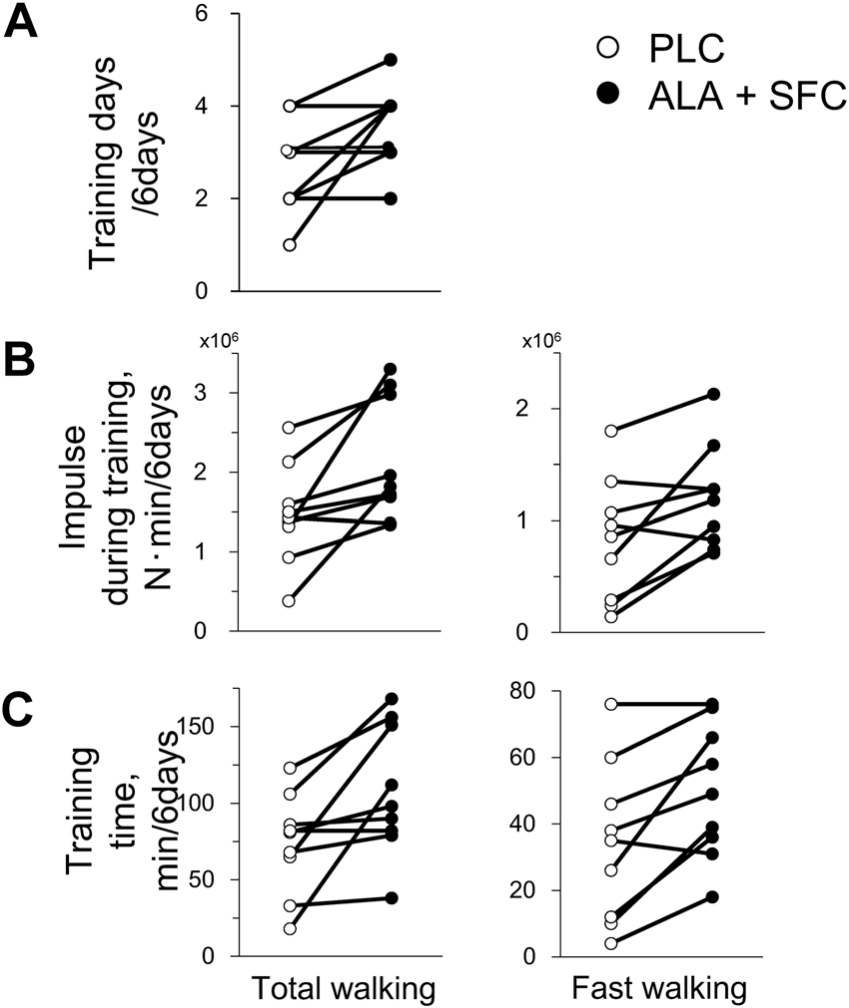
Training days (**A**), training impulse (**B**), and training time (**C**) for total and fast walking during a supplement intake period. Individual values are presented for 9 subjects.

The training days (A), training impulse (B), and training time (C) during the supplement intake period (*days 1–6*) are shown in group mean (Fig. 3) and individual values (Fig. 4). As shown in Fig. 3, training days were 33% greater in the ALA + SFC trial than the PLC trial (*P* = 0.035, 1-β = 0.602). The impulses for total and fast walking were both 46% higher in the ALA + SFC trial than the PLC trial (*P* = 0.016 and 0.014, 1-β = 0.765 and 0.794, respectively). The training times for total and fast walking were 47% and 46% higher, respectively, in the ALA + SFC trial than the PLC trial (*P* = 0.022 and 0.009, 1-β = 0.703 and 0.859, respectively).

### MADRS

**Figure 5:**
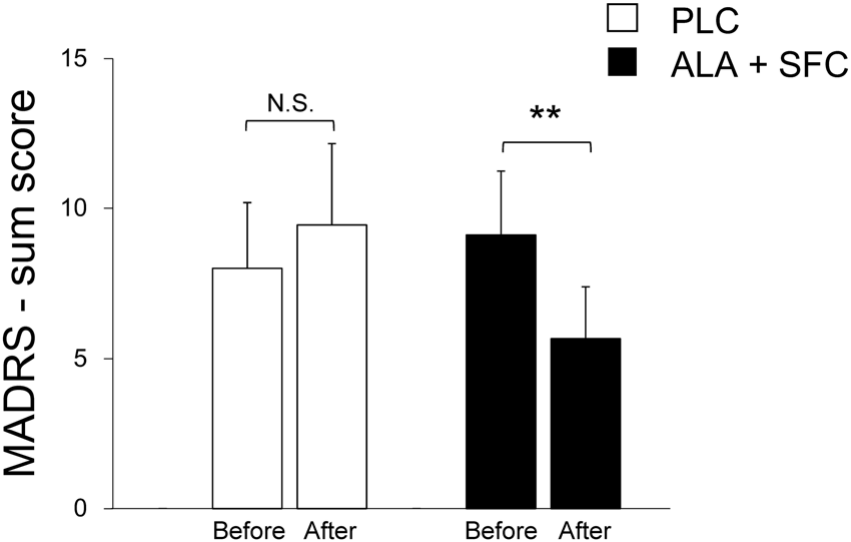
Montgomery–Åsberg Depression Rating Scale (MADRS) sum score in the PLC trial (*left*) and ALA + SFC trial (*right*), before and after a supplement intake period. Values are the means ± SE for 9 subjects. **P < 0.01 vs. before the supplement intake period.

As shown in Fig. 5, the Montgomery–Åsberg Depression Rating Scale (MADRS)^21^ score decreased significantly in the ALA + SFC trial (*P* = 0.0013, 1-β = 0.992) but not in the PLC trial (*P* > 0.1).

### Crossover analysis

Table 3 shows the results of crossover analysis. We found significant supplement effects on the average changes in ${\dot{{\rm{V}}}{\rm{O}}}_{2}$, ${\dot{{\rm{V}}}\text{CO}}_{2}$, and [Lac^−^]_p_ over the intensities ≥45 W, and training achievements, training days, impulse and time for fast walking, and the MADRS score with no carryover or period effects^22^.

**Table 3:** Crossover analysis.

		Period 1	Period 2	Carryover effect	Period effect	Supplement effect	
				P value*	P value*	Periods 1–2	P value*
Δ$\dot{{\rm{V}}}$O_2_, l/min	S1	0.01 ± 0.02	−0.00 ± 0.03	NS	NS	0.02 ± 0.02	*0.022*
	S2	−0.04 ± 0.03	0.01 ± 0.02			−0.05 ± 0.01	
Δ$\dot{{\rm{V}}}$CO_2_, l/min	S1	0.06 ± 0.03	−0.00 ± 0.03	NS	NS	0.06 ± 0.03	*0.010*
	S2	−0.05 ± 0.01	0.00 ± 0.03			−0.05 ± 0.02	
Δ[Lac^−^]_p_, mmol/L	S1	−0.1 ± 0.3	−0.6 ± 0.2	NS	NS	0.5 ± 0.3	*0.044*
	S2	−0.3 ± 0.2	0.1 ± 0.1			−0.5 ± 0.3	
Training days, days	S1	2.8 ± 0.3	3.0 ± 0.4	NS	NS	−0.3 ± 0.3	*0.032*
	S2	4.0 ± 0.3	2.6 ± 0.6			1.4 ± 0.5	
Impulse at fast walking, N min × 10^6^	S1	0.8 ± 0.2	1.1 ± 0.1	NS	NS	−0.3 ± 0.2	*0.021*
	S2	1.3 ± 0.3	0.9 ± 0.3			0.5 ± 0.2	
Time at fast walking, min	S1	32 ± 8	44 ± 6	NS	NS	−12 ± 7	*0.015*
	S2	54 ± 12	36 ± 14			19 ± 7	
ΔMADRS	S1	0.3 ± 0.5	−4.3 ± 1.4	NS	NS	4.5 ± 1.9	*0.007*
	S2	−2.8 ± 0.6	2.4 ± 1.6			−5.2 ± 1.8	

$\Delta {\dot{{\rm{V}}}{\rm{O}}}_{2}$, $\Delta {\dot{{\rm{V}}}\text{CO}}_{2}$, Δ[Lac^−^]_p_, and ΔMADRS, changes in the average values of oxygen consumption rate ($\dot{{\rm{V}}}$O_2_), carbon dioxide production rate (${\dot{{\rm{V}}}\text{CO}}_{2}$), and lactate concentration in plasma ([Lac^−^]_p_) above 45 W during the graded cycling and in the Montgomery–Åsberg Depression Rating Scale (MADRS) after supplement intake compared with before supplement intake. S1, PLC-(ALA + SFC) sequence; S2, (ALA + SFC)-PLC sequence. *S1 vs. S2 for each effect. Values are the means ± SE for 4 subjects for S1 and 5 subjects for S2. We tested the effects of 3 factors: carryover (physiological and other effects of the first supplement period are still present when the subject enters the second supplement period), period (the effect of stimulation order was present in PLC-(ALA + SFC) sequence group vs. (ALA + SFC)-PLC sequence group), and supplement effects on the average changes in ${\dot{{\rm{V}}}{\rm{O}}}_{2}$, ${\dot{{\rm{V}}}\text{CO}}_{2}$, and [Lac^−^]_p_ ≥45 W during the graded cycling test (Fig. 2B). The analysis was performed by the method reported by Chow and Liu^28^. Similarly, the analysis was also performed to test the effects of 3 factors on training days, training impulse and time for fast walking during the supplement intake period and on the MADRS score.

## Discussion

To our knowledge, this is the first study to investigate the effects of nutritional supplements on [Lac^−^]_p_ during exercise and home-based walking training achievement in depressive patients. The major findings of this study are 1) the increases in [Lac^−^]_p_, ${\dot{{\rm{V}}}{\rm{O}}}_{2}$ and ${\dot{{\rm{V}}}\text{CO}}_{2}$ during the graded cycling test were significantly suppressed in the ALA + SFC trial compared with in the PLC trial; 2) training days, training impulse, and time for fast walking significantly increased in the ALA + SFC trial compared with the PLC trial; and 3) the MADRS score was significantly decreased only in the ALA + SFC trial in middle-aged depressive women.

As shown in Fig. 2, increases in ${\dot{{\rm{V}}}{\rm{O}}}_{2}$, ${\dot{{\rm{V}}}\text{CO}}_{2}$, and [Lac^−^]_p_ during the graded cycling test were attenuated in the ALA + SFC trial but not in the PLC trial with no carryover or period effects (Table 3). Masuki *et al*.^15^ reported similar results as in the present study in older women aged ~65 years old with no depression who had performed IWT for >12 months before participating in the study, assuming that their respiratory and [Lac^−^]_p_ responses to graded cycling exercise had reached a steady state. They suggested that the increases in ${\dot{{\rm{V}}}{\rm{O}}}_{2}$, ${\dot{{\rm{V}}}\text{CO}}_{2}$, and [Lac^−^]_p_ during the graded cycling test were attenuated in the ALA + SFC trial. Regarding the mechanisms, they suggested that ALA + SFC supplementation improves mitochondrial functions to recover the age-associated decrease in transient O_2_ utilization rates for aerobic ATP production^23,24^, as well as exercise efficiency determined as work per the total metabolic cost of exercise, to reduce the O_2_ deficit at the onset of exercise. In the present study, although the subjects were ~10 years younger than those in the previous study, ${\dot{{\rm{V}}}{\rm{O}}}_{2}$_peak_ (Table 2) and probably mitochondrial function were reduced to a similar level as those in older subjects in the previous study^15^, which might have been caused by their lack of an exercise training habit and depression^25,26^. As a result, the same mechanisms likely worked in the ALA + SFC trial in the present study.

As shown in Fig. 3, training days, impulse, and time for 6 days in the ALA + SFC trial were significantly higher than those in the PLC trial with no carryover and period effects (Table 3), which are findings that are consistent with the previous study^15^. Regarding the mechanisms for the increased achievement for the ALA + SFC trial, since ${\dot{{\rm{V}}}{\rm{O}}}_{2}$ and ${\dot{{\rm{V}}}\text{CO}}_{2}$ were saved above the intensities ≥45 W during graded cycling exercise after supplement intake and since the increase in [Lac^−^]_p_ was significantly attenuated above the intensity, subjective feeling for fast walking might be improved due to reduced panting and muscle pain^27,28^, ultimately resulting in increases in impulse and time at fast walking in the trial.

As shown in Fig. 5, the MADRS score significantly decreased only in the ALA + SFC trial with no carryover and period effects (Table 3), in which subjects performed fast walking for IWT for ~50 min on average for 6 days, which agrees with previous studies^7^ suggesting a moderate or higher intensity of aerobic exercise (60–80% HR_peak_), 3 days/week, for 8 weeks improved depressive symptoms. Although the training period in the present study was shorter than in previous studies^7^, Dimeo *et al*.^29^ suggested that aerobic training at a moderate exercise intensity (Borg scale 13–14), which was 30 min/day for 10 days, significantly decreased the depression scores, the Hamilton Rating Scale by 33% and the self-assessed intensity score by 24%. Moreover, it was suggested that only one bout of high intensity exercise improved mood in depressive patients^30^ with an increase in the serum concentration of brain-derived neurotrophic factor (BDNF)^31^, which is suggested to decrease in depressive patients and increase when the symptoms are improved with anti-depressant drug administration^32^. In contrast, in the PLC trial, there was no significant change before and after the supplement intake period; rather, it tended to increase the MADRS score. This finding might be due to insufficient training achievement. Alternatively, the required exercise intensity might be too difficult for depressive patients. Indeed, Weinstein *et al*.^33^ suggested that when the demanded exercise intensity is perceived by depressive patients as being too hard, it evokes a negative mood. Therefore, ALA + SFC supplementation might lower the physical and psychological barriers to achieving moderate or higher intensity exercise training to decrease the MADRS score.

There are three experimental considerations that deserve additional discussion. First, we could not exclude any direct effects of the ALA + SFC supplementation on the central nervous system related to depression mechanisms. The association between depression and mitochondrial dysfunction in various brain regions has been suggested^34^. For example, Omori *et al*.^35^ suggested that brain mitochondrial activity was enhanced by the 6-month administration of ALA in a mouse model of Alzheimer’s disease. Additionally, Perez *et al*.^36^ suggested that ALA + SFC supplementation of ~50 mg per day for 3 weeks improved the sleep quality score by ~30% (Pittsburgh Insomnia Rating Scale-20 question) in middle-aged and older people. Thus, ALA + SFC supplementation likely improves depression by activating mitochondrial function in the brain; however, since previous studies did not report physical activity during supplementation, it remains unclear how enhanced physical activity by ALA + SFC supplementation was involved in the results. In the present study, we found that the supplementation increased physical activity and improved depressive symptoms. Second, as shown in Table 2, the baseline HR_rest_ significantly decreased in the ALA + SFC trial. Since sympathetic nervous system (SNS) activity is reportedly elevated in depressive patients^37^, the improved symptoms in the trial might decrease HR_rest_. Third, this study was conducted using only 9 subjects with a relatively short supplement intake period. Based on the present findings, a larger and longer trial to examine the effects of this treatment will be needed.

In summary, ALA + SFC supplementation improved respiratory and [Lac^−^]_p_ responses during high-intensity exercise, increased fast-walking training achievement, and improved symptoms in middle-aged women with depression.

## Methods

### Subjects

This study protocol was approved by the Review Board on Human Experiments, Shinshu University School of Medicine, and conformed to the standards set by the Declaration of Helsinki. The trial was registered in UMIN (trial registration number: UMIN000013210) on February 21, 2014.

We recruited female subjects aged 40–70 years who had no exercise habit from outpatients visiting our clinic in Tokyo for depression using a pamphlet at their scheduled examination. The recruitment was performed from February 22 to September 30, 2014. The inclusion criteria were that (1) they were diagnosed with major depressive disorder according to the Diagnostic and Statistical Manual of Mental Disorder (DSM ver.4)^38^ and had received psychotropic medication and psychotherapy, and the treatments were stabilized for 8 weeks; (2) they had recently experienced no drastic lifestyle changes; and (3) they were non-smokers and had no overt history of orthopaedic diseases to disturb IWT and iron deficiency anaemia to influence the results. We recruited female subjects to minimize any confounding effects of gender.

Eleven of 17 responders provided written informed consent and agreed to participate in this study. Since 2 subjects did not complete the protocol of the graded cycling test, we analysed the results in the remaining 9 subjects. However, no harmful events occurred during the intervention.

### Randomization

Subjects were randomly assigned to PLC-(ALA + SFC) sequence or (ALA + SFC)- PLC sequence by an independent investigator (K.H.) using permuted-block randomization (block size: 4) with an allocation ratio of 1:1. The investigator was not involved in participant recruitment or any assessments. The random allocation sequence was generated using a computer.

### Protocol

This study was carried out from February 22 to December 9, 2014 in a randomized, placebo-controlled, double-blind crossover design **(**Fig. 1**)** with an allocation ratio of 1:1. All subjects participated in two trials for 9 days each, followed by ≥10-day washout period; 7 days (*days 1–7*) for supplement intake and 2 days for graded cycling tests before and after the trial (*days 0 and 8*). Subjects consumed either ALA + SFC (ALA + SFC trial) or the placebo supplement (PLC trial) for ≥1 hr before breakfast and dinner. During the graded cycling test, the cardiorespiratory responses and [Lac^−^]_p_ were measured. During *days 1–6*, the training days, intensity, and time were recorded with a tri-axial accelerometer (JD Mate; Kissei Comtec, Matsumoto, Japan)^39,40^. There was no training on *day 7* to avoid any acute influence of IWT on the graded cycling test. In subjects who still had a menstrual cycle, the experiments were scheduled during their follicular phases.

### Depression severity

Before the graded cycling exercise test, a psychiatrist examined depression severity using the MADRS^21^; he was unaware of whether the subjects belonged to the ALA+ SFC or PLC trials.

### Supplements

The composition of supplements (SBI ALApromo, Tokyo) is shown in Supplemental Table S1. The dose of ALA phosphate (100 mg/day) was the same as in the previous study using older women with no depression^15^. SFC, as a source of the iron ion in the supplements, was used to enhance the final step of haeme biosynthesis by the ABCB6 transporter and ferrochelatase in mitochondria^41^. The ALA + SFC and placebo supplements were similar in appearance, and all of the subjects and investigators who performed experiments and analyses were blinded to which trial the subjects actually underwent until all of the analyses were finished.

### Dietary intake

Subjects in both trials were instructed to maintain their dietary habits including medications, except for the supplements, throughout the study while reporting food consumed during the period by answering a questionnaire prepared by a dietician (FFQg version 4.0; Kenpakusya, Tokyo). We confirmed no significant differences in the values between the trials, including n-3 polyunsaturated fatty acid, which was reported to improve depressive symptoms^42^ (all, *P* > 0.4). Moreover, the amount of ALA contained in the diet^43^ was negligible compared to that in the supplement (Supplemental Tables S1, S2).

### Graded cycling test

To minimize any inter-individual variation by different levels of food intake on the graded cycling test, we provided the standardized meals on the day before the test. At 0900 on the morning of the test, the subjects reported to the clinic that they were normally hydrated but had not eaten any food for more than 12 h before the experiment, except for a supplement 2 h before the visit. After measuring anthropological variables, the subjects entered a laboratory in the clinic controlled at ~25 °C and ~40% relative humidity. After a Teflon catheter was placed in the antecubital vein for blood sampling, the subjects rested quietly in an upright position on the saddle of the cycle ergometer for 15 min while all measurement devices were applied. After 10 min at rest, subjects started the cycling exercise at 60 revolutions/min at 0 W for 3 min, and then the intensity was increased to 15 W; then, the intensity was increased by 15 W every 2 min until the subjects were exhausted, during which time ${\dot{{\rm{V}}}{\rm{O}}}_{2}$, ${\dot{{\rm{V}}}\text{CO}}_{2}$, and ${\dot{{\rm{V}}}}_{{\rm{E}}}$ were measured with a respiratory gas analyser (Metamax3B; Cortex, Leipzig, Germany) (Fig. 2, Table 3**)** and heart rate (HR) was measured with a pulse rate monitor (Polar RS400; Vantage NV, Kempele, Finland) **(**Table 2**)**. The criteria for judging whether exercise intensity reached ${\dot{{\rm{V}}}{\rm{O}}}_{2}$_peak_ were that (1) subjects were not able to maintain the rhythm, (2) the respiratory quotient increased to over 1.1, and (3) HR reached the age-predicted maximal value. The ${\dot{{\rm{V}}}{\rm{O}}}_{2}$_peak_ was determined by averaging the highest three consecutive values at the end of the exercise.

### Blood samples

Blood samples were taken at rest and at the last minute of each intensity to determine [Lac^−^]_p_ (YSI 2300 Stat Plus; Yellow Springs, OH) (Fig. 2, Table 3**)** and [Hb] at rest (Microsemi LC-660; HORIBA, Tokyo).

### Training achievement

During the supplement intake period, except for the day before the second graded cycling test (*days 1–6*), subjects were instructed to perform IWT with the goal of repeating ≥5 sets of 3 min of slow walking at 40% $\dot{{\rm{V}}}$O_2 peak_, followed by 3 min of fast walking above 70% ${\dot{{\rm{V}}}{\rm{O}}}_{2}$_peak_ per day, for ≥4 days/wk; during this period, the intensity and duration were recorded with a portable tri-axial accelerometer (JD Mate)^39,40^, and the measurements were transferred to the server computer at Shinshu University through the Internet after training^18–20^. Training intensity was calculated from the product of body weight and average norm of three-dimensional accelerations and presented as the accumulated training impulse (N·min)^39,40^ for 6 days (Fig. 3).

### Analyses

#### ${\dot{{\rm{V}}}{\rm{O}}}_{2}$, ${\dot{{\rm{V}}}\text{CO}}_{2}$, and [Lac^−^]_p_ at the intensity ≥45 W

As shown in Fig. 2B, we analysed the average values of ${\dot{{\rm{V}}}{\rm{O}}}_{2}$, ${\dot{{\rm{V}}}\text{CO}}_{2}$, and [Lac^−^]_p_ over the intensities ≥45 W in each subject for each trial since the increase in [Lac^−^]_p_ was significantly attenuated above the intensity in the ALA + SFC trial (Fig. 2A)_._

### Statistics

Adequate sample size was determined based on the previous study assessing the effects of ALA + SFC supplementation on IWT achievements and ${\dot{{\rm{V}}}{\rm{O}}}_{2}$, ${\dot{{\rm{V}}}\text{CO}}_{2}$, and [Lac^−^]_p_ during exercise in older women^15^ and the statistical power calculation.

One-way ANOVA for repeated measures was used to examine any significant differences in physical characteristics before vs. after the supplement intake period (Table 2) and examine any significant differences in dietary intake for the period between trials (Supplemental Table S2). This model was also used to examine any significant differences in training days, training impulse, and training time during the supplement intake period (Fig. 3); in the MADRS score before and after the period (Fig. 5); and in changes in ${\dot{{\rm{V}}}{\rm{O}}}_{2}$, ${\dot{{\rm{V}}}\text{CO}}_{2}$, and [Lac^−^]_p_ ≥45 W (Fig. 2B) after the period between trials. Two-way ANOVA for repeated measures was used to examine any significant differences in the variables at every intensity during the graded cycling test before vs. after the supplement intake period in each trial, with a significant interactive effect of [(before vs. after the supplement intake period) × time] (Fig. 2A). As a subsequent post hoc test, the Tukey-Kramer test was used to perform any pairwise comparisons between trials.

In addition, because this study was conducted in a two-period crossover design, we performed a crossover analysis to examine the effects of 3 factors^22^ – carryover, period, and supplements – on the results, as described in the caption in Table 3.

The statistical power (1-β) is presented in the text as α = 0.05 when the key variables were significantly different between the PLC and ALA + SFC trials^44^. The null hypothesis was rejected when *P* < 0.05. Values are expressed as the means ± SE, unless otherwise indicated.

### Data Availability

The datasets generated during and/or analysed during the current study are available from the corresponding author on reasonable request.

## Electronic supplementary material

Supplemental_Tables 1 and 2
