# Supplementary material for: Effects of 5-aminolevulinic acid supplementation on home-based walking training achievement in middle-aged depressive women: randomized, double-blind, crossover pilot study
Source: Sci Rep. 2018 May 8;8:7151. doi: 10.1038/s41598-018-25452-2 (PMC5940675; doi:10.1038/s41598-018-25452-2)
Supplement: Supplementary file 1 — Supplemental_Tables 1 and 2 [file 41598_2018_25452_MOESM1_ESM.doc]

**Effects of 5-aminolevulinic acid supplementation on home-based walking training achievement in middle-aged depressive women: randomized, double-blind, crossover pilot study**

Hiroshi Suzuki^1,3,4^, Shizue Masuki^1,2^, Akiyo Morikawa^4^, Yu Ogawa^1^, Yoshi-ichiro Kamijo^1,2^, Kiwamu Takahashi^5^, Motowo Nakajima^5^, and Hiroshi Nose^1,2^

^1^Dept. of Sports Med. Sci., Shinshu Univ. Grad. Sch. of Med., ^2^Inst. for Biomed. Sci., Shinshu Univ., Matsumoto 390-8621; ^3^Aoba Kokoro-no Clinic, ^4^Aoba Promotion Co., Ltd., Tokyo 170-0002; and ^5^Dept. of R&D, SBI Pharmaceuticals Co., Ltd., Tokyo 106-6020, Japan

| **Supplemental Table S1:** *Composition of supplements* | |  |
| --- | --- | --- |
|  | Placebo Supplement (250.00 mg/dose) | ALA+SFC  Supplement  (250.00 mg/dose) |
| ALA phosphate, mg | 0.00 | 50.00 |
| SFC, mg | 0.00 | 57.36 |
| Pre-gelatinized starch, mg | 247.50 | 140.14 |
| Silicon dioxide mixture, mg | 2.50 | 2.50 |

ALA, 5-aminolevulinic acid; SFC, sodium ferrous citrate

| **Supplemental Table S2:** *Food intake by diet without supplements per day during a training period* | | |
| --- | --- | --- |
|  | PLC | ALA+SFC |
| Energy, kcal | 1597 ± 88 | 1575 ± 102 |
| Protein, g | 60.2 ± 4.4 | 59.3 ± 4.0 |
| Fat, g | 53.7 ± 4.6 | 55.0 ± 4.8 |
| Carbohydrate, g | 211.4 ± 9.5 | 205.5 ± 12.1 |
| ALA, µg | 42.9 ± 3.1 | 43.9 ± 3.2 |
| Iron, mg | 6.2 ± 0.5 | 5.9 ± 0.4 |
| n-3 polyunsaturated fatty acid, g | 1.9 ± 0.2 | 1.9 ± 0.2 |

PLC, placebo intake condition; ALA+SFC, 5-aminolevulinic acid + sodium ferrous

citrate intake condition; values are the means ± SE for 9 subjects in each trial.
